# Trends in State Palliative Care Legislation Across the US

**DOI:** 10.1001/jamahealthforum.2025.4731

**Published:** 2025-10-24

**Authors:** Na Ouyang, Ling Han, Wendy Jiang, Stacie Sinclair, Eugene Rusyn, Shelli L. Feder

**Affiliations:** 1 Yale School of Nursing, Orange, Connecticut; 2 Department of Internal Medicine, Yale School of Medicine, New Haven, Connecticut; 3 Yale Law School, New Haven, Connecticut; 4 Center to Advance Palliative Care, New York, New York; 5 VA Connecticut Healthcare System, West Haven

## Abstract

This cross-sectional study examines trends in the introduction and enactment of state-level palliative legislation, categorizes legislative content, and maps distribution across states and regions from 2009 to 2023.

## Introduction

More than 13.7 million people in the US could benefit from palliative care.^1^ Yet, access is uneven due to workforce shortages, low public awareness, variability in service availability, and federal delays.^2^ Although states play a pivotal role in shaping health policy, the extent of their legislative efforts regarding palliative care is unknown. Using data from the Palliative Care Law and Policy GPS, a database developed by the Yale Solomon Center for Health Law and Policy in partnership with the Center to Advance Palliative Care,^3^ we examined trends in the introduction and enactment of state-level palliative legislation, categorized legislative content, and mapped distribution across states and regions from 2009 to 2023.

## Methods

This cross-sectional study adhered to EQUATOR reporting guidelines and did not involve human participants. The GPS tracks state legislation related to palliative care identified through keyword searches using Lexis+, LegiScan, and State Net.^3^ Legislation is classified into 8 nonmutually exclusive categories: clinical skill building, patient rights/protections, payment, pediatric palliative and hospice care, public awareness, quality/standards, telehealth, and workforce (eMethods in Supplement 1). Data were abstracted from the GPS from 2009 to 2023. Two authors (W.J. and E.R.) reviewed and extracted relevant legislation, counting identical bills once per session and treating different bills as separate entries.

We used R, version 4.0.2 (R Project for Statistical Computing), and Excel 2024 (Microsoft) to generate descriptive statistics by year, category, state, and region. We calculated yearly and total counts of introduced and passed bills, along with crude pass-to-introduced ratios and pass rates by introduction frequency.

## Results

**Figure 1.**
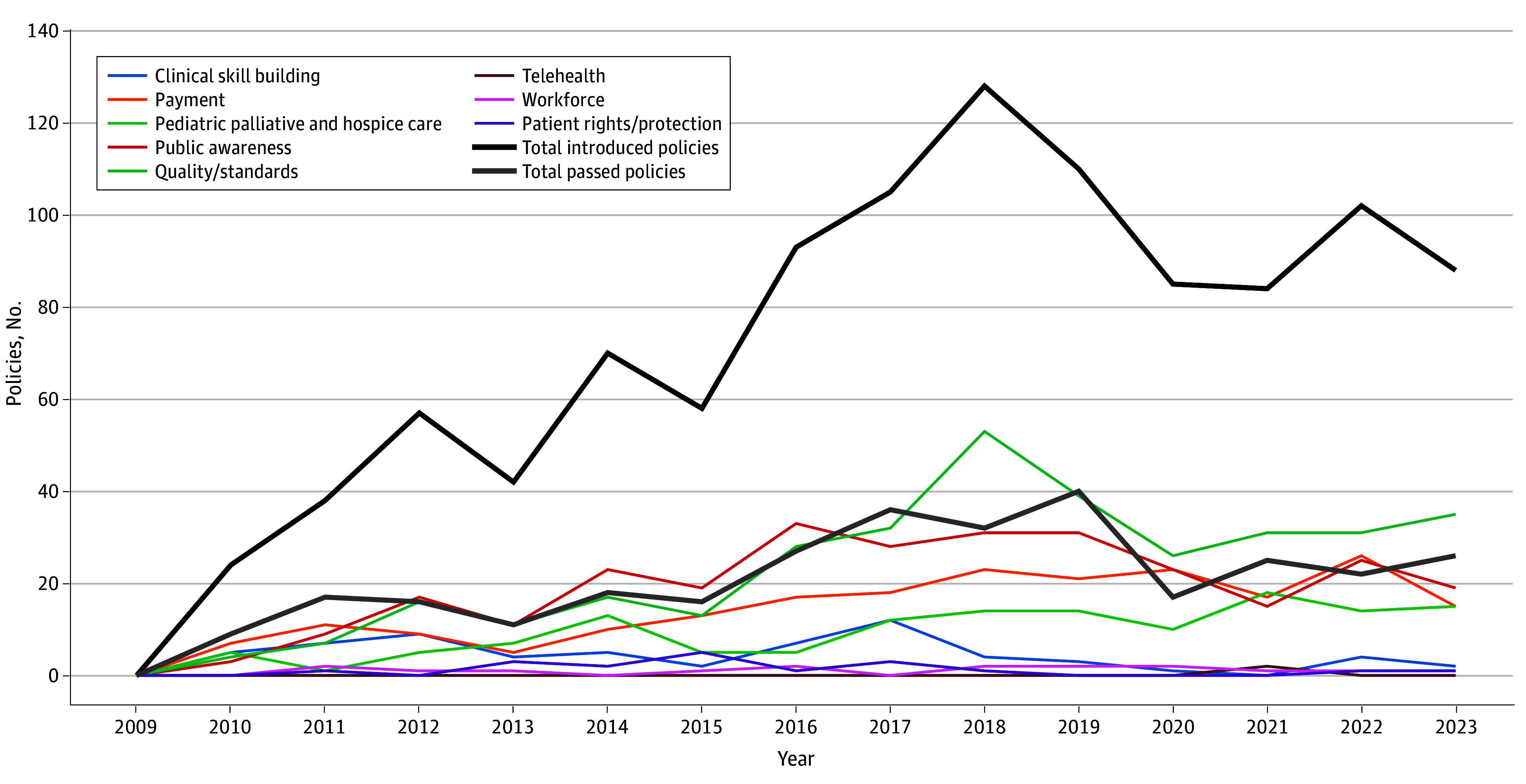
Number of Palliative Care Bills Introduced in the US by Category This line graph displays the number of palliative care–related policies introduced each year across 8 policy categories. Colored lines represent different policy categories, while the black line indicates the total number of policies introduced annually, and the gray line indicates the total number of policies passed.

States introduced 819 pieces of legislation during the study period, peaking in 2018. States most frequently introduced quality/standards, public awareness, and payment policies (Figure 1). States that introduced the most legislation included Massachusetts with 111 pieces, New York with 72, and New Jersey with 61.

**Figure 2.**
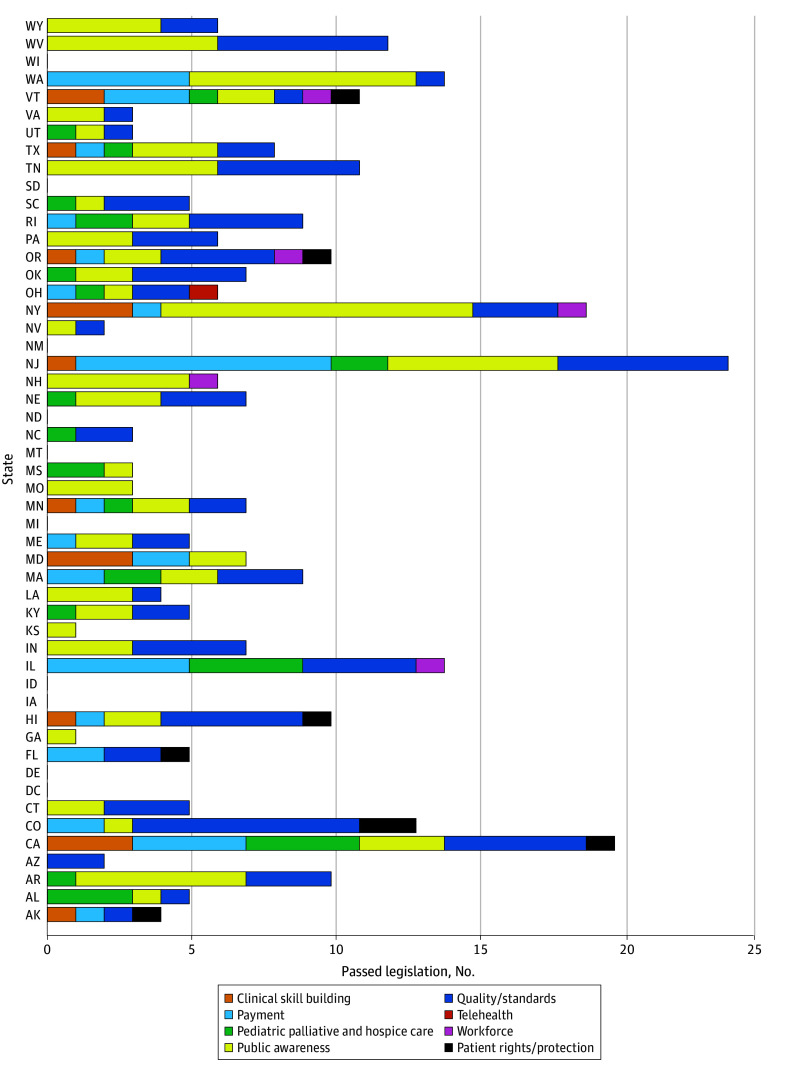
Number of Bills Passed by Category Across States This horizontal stacked bar chart shows the number of passed palliative care bills in each US state from 2009 to 2023, categorized by policy focus. Each color represents a different policy category.

Passed legislation followed a similar pattern, peaking in 2019. States that enacted the most legislation included New Jersey with 18 pieces and New York with 12. Most states passed palliative legislation related to quality/standards and public awareness (Figure 2). Few states passed legislation on telehealth, workforce, and patient rights/protections. The Northeast passed the most legislation, with 101 pieces, and the Midwest passed the least legislation, with 45 pieces, during the study period.

Crude pass rates were 29.8% (range, 20.6%-42.9%). Of 723 unique bills, 669 (92.5%) were introduced once, and of 243 enacted legislation pieces, 236 (97.1%) passed on first introduction. Passage rates declined with each repeated introduction; pass rates were 35.5% for legislation introduced once, dropping to 19.3% for legislation introduced twice.

## Discussion

This cross-sectional study demonstrates that state palliative care laws have increased in the past 15 years, but policy and regional gaps persist. Most introduced and enacted policies focused on quality/standards, public awareness, and payment. Notably, most enacted legislation passed on first introduction. Success rates declined sharply with repeated attempts, highlighting that political feasibility is often established early in the legislative cycle. Study limitations include the potential for missed or misclassified palliative policies and the exclusion of state regulations, which warrants future research.

Substantial regional disparities in legislative activity likely reflect broader inequities in policy engagement, as states with higher legislative activity typically have stronger palliative care capacity.^4^ Legislative trends found in this report align with higher-performing states in the recent Serious Illness Scorecard from the Center to Advance Palliative Care, which measures each state’s capacity to advance palliative care initiatives. Two out of 10 Serious Illness Scorecard measures examine legislative activity related to payment and public awareness, which may partly explain this alignment. States with more building blocks may be better equipped to pass further legislation. This study highlights broad trends in palliative care policymaking, emphasizing opportunities for advancing palliative care policy through targeted advocacy and research.

## Notes

Supplement 1.
eMethods

Supplement 2.
Data Sharing Statement
