# Error in Figure 1

**DOI:** 10.1001/jamahealthforum.2025.5878

**Published:** 2025-12-05

The Research Letter titled “Trends in State Palliative Care Legislation Across the US,”^1^ published online October 24, 2025, contained an error in Figure 1. There was a total of 9 passed policies for 2010. This article was corrected online.
