# Supplementary material for: Trends in State Palliative Care Legislation Across the US
Source: JAMA Health Forum. 2025 Oct 24;6(10):e254731. doi: 10.1001/jamahealthforum.2025.4731 (PMC12552922; doi:10.1001/jamahealthforum.2025.4731)
Supplement: Supplement 1. — eMethods [file jamahealthforum-e254731-s001.pdf]

Supplemental Online Content

Ouyang N, Han L, Jiang W, Sinclair S, Rusyn E, Feder SL. Trends in state palliative care legislation across the US. *JAMA Health Forum*. 2025;6(10):e254731. doi:10.1001/jamahealthforum.2025.4731

eMethods

This supplemental material has been provided by the authors to give readers additional information about their work.

© 2025 Ouyang N et al. *JAMA Health Forum*.

eMethods

The Palliative Care Law and Policy GPS (PCLP GPS) is a publicly available database developed by the Solomon Center for Health Law and Policy at Yale Law School in partnership with the Center to Advance Palliative Care (CAPC). The GPS compiles state legislation related to palliative care identified through keyword searches (“palliative” or “palliative care”) using Lexis+, LegiScan, and StateNet. Policies focused exclusively on end-of-life, hospice care, medical marijuana, and psilocybin are excluded from the GPS to ensure emphasis on upstream palliative care delivery.

Data for this analysis were abstracted from the GPS from 2009 to 2023. Abstracted data included state, title of a piece of legislation, year of introduction and passage (or last action), legislative summary, and category. The research team identified eight non-mutually exclusive policy categories during the initial creation of the GPS by reviewing resources relevant to state-level palliative care policy, including the CAPC and the National Palliative Care Research Center State-by-State Palliative Care Report Card (Report Card), and reports by the National Academy for State Health Policy. These eight categories are clinical skill-building, patient rights/protections, payment, pediatric palliative and hospice care, public awareness, quality/standards, telehealth (specific to palliative care), and workforce.

Two authors extracted palliative care legislation from the GPS and created a longitudinal dataset reviewing full bill texts to confirm relevance. Where more than one bill related to the same substantive text (if, for instance, a bill was introduced in both houses of a state legislature) in the same legislative session, the text of each bill was compared against the other using track changes. If there were no substantive changes to the text specifically related to palliative care between the bills, they were tallied as one entry to avoid overcounting the number of relevant legislation. Where there were substantive differences in the text, the bills were treated as distinct.

© 2025 Ouyang N et al. *JAMA Health Forum*.
