# Supplementary material for: Trends in State Palliative Care Legislation Across the US
Source: JAMA Health Forum. 2025 Oct 24;6(10):e254731. doi: 10.1001/jamahealthforum.2025.4731 (PMC12552922; doi:10.1001/jamahealthforum.2025.4731)
Supplement: Supplement 2. — Data Sharing Statement [file jamahealthforum-e254731-s002.pdf]

Data Sharing Statement

Ouyang. Trends in State Palliative Care Legislation Across the US. *JAMA Health Forum*. Published October 24, 2025. doi:10.1001/jamahealthforum.2025.4731

Data
Data available: Yes
Data types: Data (not involving human participants)
How to access data: The raw data are available at: https://palliativecarelawandpolicy.yale.edu/
When available: With publication

Supporting Documents
Document types: None

Additional Information
Who can access the data: It will be available to anyone requesting the data.
Types of analyses: For any purpose
Mechanisms of data availability: The GPS is publically available.
